# The missing link: does tunnelling nanotube-based supercellularity provide a new understanding of chronic and lifestyle diseases?

**DOI:** 10.1098/rsob.160057

**Published:** 2016-06-08

**Authors:** Amin Rustom

**Affiliations:** Interdisciplinary Center for Neurosciences (IZN), Institute of Neurobiology, University of Heidelberg, INF 364, 69120 Heidelberg, Germany

**Keywords:** tunnelling nanotube, heteroplasmy, reactive oxygen species, oxidative stress, plasmodesmata, supercellularity

## Abstract

Tunnelling nanotubes (TNTs) are increasingly recognized as central players in a multitude of cellular mechanisms and diseases. Although their existence and functions in animal organisms are still elusive, emerging evidence suggests that they are involved in developmental processes, tissue regeneration, viral infections or pathogen transfer, stem cell differentiation, immune responses as well as initiation and progression of neurodegenerative disorders and cancer (see Sisakhtnezhad & Khosravi 2015 *Eur. J. Cell Biol.* 94, 429–443. (doi:10.1016/j.ejcb.2015.06.010)). A broader field of vision, including their striking functional and structural resemblance with nanotube-mediated phenomena found throughout the phylogenetic tree, from plants down to bacteria, points to a universal, conserved and tightly regulated mechanism of cellular assemblies. Based on our initial definition of TNTs as open-ended channels mediating membrane continuity between connected cells (Rustom *et al.* 2004 *Science* 303, 1007–1010. (doi:10.1126/science.1093133)), it is suggested that animal tissues represent supercellular assemblies that—besides opening discrete communication pathways—balance diverse stress factors caused by pathological changes or fluctuating physiological and environmental conditions, such as oxidative stress or nutrient shortage. By combining current knowledge about nanotube formation, intercellular transfer and communication phenomena as well as associated molecular pathways, a model evolves, predicting that the linkage between reactive oxygen species, TNT-based supercellularity and the intercellular shuttling of materials will have significant impact on diverse body functions, such as cell survival, redox/metabolic homeostasis and mitochondrial heteroplasmy. It implies that TNTs are intimately linked to the physiological and pathological state of animal cells and represent a central joint element of diverse diseases, such as neurodegenerative disorders, diabetes or cancer.

## 1. Introduction

Since our discovery of tunnelling nanotubes (TNTs) in 2004 [1], the phenomenon of ‘nanotubular’ cell connections has gained a lot of attention. However, only a handful of studies address this topic in the *in vivo* situation [2], e.g. for myeloid cells in mouse cornea [3] and human lung adenocarcinoma tumour cells [4,5]. Furthermore, in most TNT-related studies the differentiation between—*per definitionem*—open-ended TNTs and close-ended membrane tubes sharing a similar appearance, such as filopodia, retraction fibres, cytonemes [6], tumour microtubes [7], streamers [8], etc., appears as an unsolvable task. Thus, until now, and in contrast with e.g. plant plasmodesmata, the existence of open-ended membrane channels as a general principle of supercellularity has not been unequivocally proven for animal cells in direct contact, not to speak from the *in vivo* situation.

Nevertheless, numerous *in vitro* studies analysing dispersed cell systems [2], including primary cultures and tissue explants, suggest that there is a direct link between nanotube formation, intercellular material transfer and detrimental physiological, pathological and environmental conditions, such as oxidative stress or metabolic strains. In this context, hydrogen peroxide (H_2_O_2_) increased the number of TNTs in co-cultures of rat primary astrocytes and C6 glioma cells [9] and the amount of TNT-like intercellular connections in rat primary astrocytes [10]. Likewise, TNT development and bidirectional transfer of vesicles, proteins and mitochondria can be induced in human malignant pleural mesothelioma by serum depletion [4]. Increased nanotube numbers and attenuated kidney tissue damage were also observable in murine kidney tissue under elevated oxidative stress, such as renal ischaemia or peritoneal dialysis [11]. It is intriguing to note that similar observations were made in plant systems, where reactive oxygen species (ROS) lead to enhanced formation of secondary plasmodesmata and increased symplastic connectivity [12].

In general, oxidative stress is defined as an imbalance between the production of free radicals and reactive metabolites, such as H_2_O_2_ or superoxide anions, and their elimination by the antioxidative cell defence system. The list of severe diseases that have been linked to oxidative stress is long, including neurodegenerative disorders, such as Alzheimer's and Parkinson's, chronic inflammation, diabetes and cancer [13]. As it is well accepted that most ROS are generated in cells by the mitochondrial respiratory chain [14], it is hardly surprising that the respective organelles are caught in the crosshairs of current medical research.

Because it was realized that functional mitochondria can be transferred via TNTs between various cell types, studies have proven that this transfer serves as a potent rescue mechanism to compensate for severe stress conditions (for review, see [2]). This allows e.g. cancer cells to survive extreme scenarios, such as the loss of mitochondrial functionality [15]. These findings complete the picture of an intimate link between ROS levels, TNT-based supercellularity and the intercellular shuttling of materials that defines the overall condition of animal cells in health and disease. Along with the assumption that TNTs are a universal feature, this linkage culminates in the following three-stage model, describing a surprisingly simple framework that could significantly update our understanding of biological and pathological processes.

## 2. Mechanistic model of reactive oxygen species-dependent tunelling nanotube formation

### 2.1. Stage 1: maintenance of redox/metabolic homeostasis

**Figure 1.**
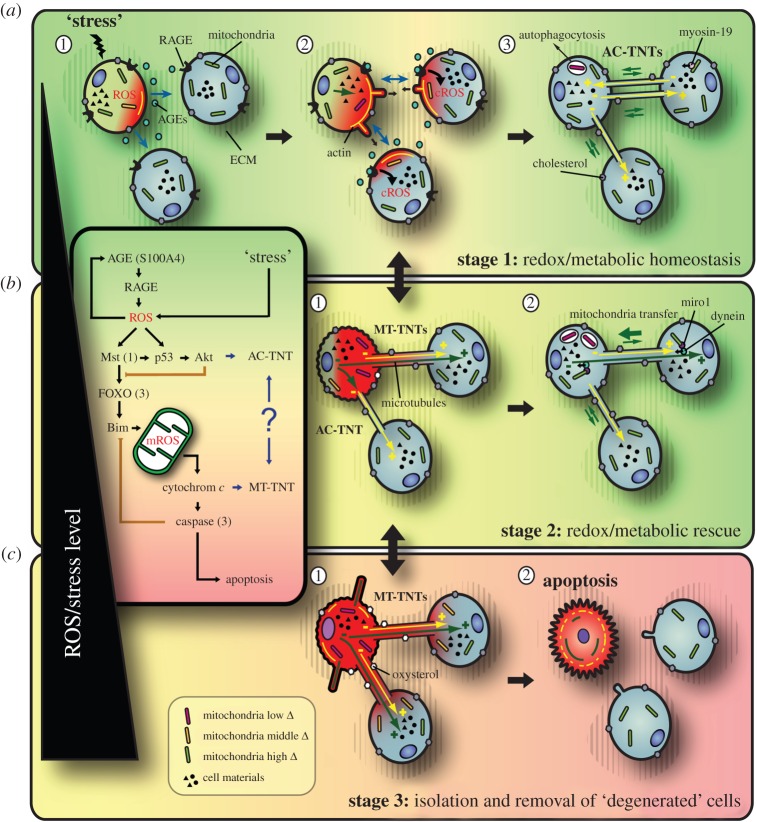
Model of ROS-dependent TNT formation: a universal principle of cellular assemblies to level out stress conditions? Local stress leads to increasing ROS levels and AGE distribution from the stressed cell (*a*-1). AGE–RAGE interaction at the target cells leads to cROS increase (*a*-2) and AC-TNT formation via actin-based, filopodia-like cell protrusions in order to restore redox/metabolic homeostasis by intercellular material exchange (*a*-3). Further increasing ROS levels lead to MT-TNT formation (*b*-1), allowing for efficient redox/metabolic rescue of stressed cells, e.g. via motor protein-mediated intercellular transfer of mitochondria along microtubules (*b*-2). Finally, exaggerated ROS levels induce apoptosis (*c*-1). Note that prior to apoptosis, remaining TNT connections are severed in order to isolate and remove ‘degenerated’ cells from the collective (*c*-2)—probably controlled by altered cholesterol/oxysterol homeostasis. Increasing ROS levels are indicated by the green to yellow to red colour gradient. The superimposed box depicts the key players of the suggested molecular pathway. Black arrows indicate positive feedback; orange blunt arrows indicate negative feedback.

Physiological, pathological and environmental influences, such as oxidative and reductive stress, hypoxia, hyperglycaemia, nutrient shortage, ultraviolet (UV) radiation, etc., can lead to local cell stress, accompanied by ROS increase (figure 1*a*-1). To counter this, stressed cells will distribute ‘call-for-help’ signals to determine the position of unstressed cells in their surroundings. Although the nature of these signals is still under debate, candidate molecules are advanced glycation end products (AGEs), such as S100A4 [16]. Indeed, high concentrations of S100A4 were shown to induce and attract outgrowing TNTs from e.g. astrocytes [16].

**Figure 2.**
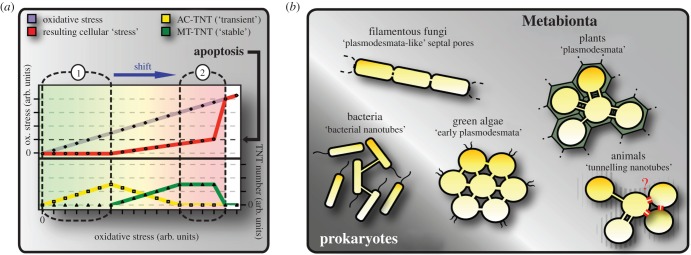
(*a*) Hypothetical correlation of AC- and MT-TNT formation with a reduction of cellular stress levels. Within a tolerance range (*a*-1) AC-TNT formation (yellow) can compensate for increasing oxidative stress (purple), keeping cellular stress levels on a constant level (red). Reaching the limit of AC-TNT-based compensation marks the onset of MT-TNT formation (green), reducing cellular stress levels. If a critical stress level is reached (black arrow), apoptosis will be initiated and remaining TNT connections severed. Note that the shift from ‘transient’ AC-TNT towards ‘stabilized’ MC-TNT formation (blue arrow) may be a common feature of chronic and lifestyle diseases, such as neurodegenerative disorders, diabetes or cancer. Increasing ROS levels are additionally indicated by the green to yellow to red colour gradient. (*b*) ‘Nanotube’-mediated supercellularity: a general principle among cellular life-forms? Some bacteria and various multicellular organisms belonging to different kingdoms exhibit a supercellular organization based on nanotubular cell connections mediating membrane continuity, pointing to the existence of a universal and conserved mechanism. Note that the general existence of TNTs in animal tissues—i.e. between cells in direct contact—so far is elusive (red). Adapted from Rustom [27].

Corresponding receptors on the target cells, here the receptor for advanced glycation end products (RAGE), will function as ‘signal receivers’ (figure 1*a*-2). AGE–RAGE interaction will lead to local cytoplasmic (c)ROS production that initiates a self-amplifying ‘ping–pong’ loop between sender and receiver, determining the optimal positions for TNT formation. Consistently, multiple studies with RAGE-expressing cells have demonstrated that ligand–RAGE interaction leads to the generation of cROS, downstream signal transduction and regulation of gene expression [17]. A comparable mechanism was observed during hyphal fusion, where components of the MAK-2 mitogen activated protein kinase cascade, e.g. Ste-50, are recruited in an oscillatory manner to the tips of communicating germlings of *Neurospora crassa* [18].

Only if a defined ROS level is reached—probably at first in the initially stressed sender cells—the formation of TNTs via actin-based filopodia-like cell protrusions will be initiated (figure 1*a*-2). Accordingly, in a RAGE(–/–) knockout mouse model under standard conditions, strongly reduced TNT numbers were detectable [11]. Also knocking down RAGE in astrocytes reduced the number of TNTs formed towards the target cells [16]. Furthermore, Chinese hamster ovary (CHO) cells, which lack endogenous RAGE, were not able to develop TNTs under normal conditions [16]. A potentially related model suggests that in TNT-initiating cells, the tumour suppressor p53 activates caspase-3, which leads to S100A4 cleavage, resulting in AGE gradients, potentially involved in TNT guidance [16]. This scenario describes an efficient way to avoid TNT formation towards stressed or pathological cells. It might be reasonably assumed that also other factors, in particular the condition of the extracellular matrix (ECM), will have significant impact on TNT formation [19]. In this context, it was shown that hyaluronan synthase 3 overexpression induces the formation of filopodia-like cell protrusions [20] resembling TNTs, a process potentially linked with the PI3K pathway [21].

On the molecular level, TNT formation is probably controlled by a ROS-dependent pathway that links AGE–RAGE signalling and redox homeostasis with cytoskeletal modifications and finally leads to apoptosis (figure 1, box). At this stage, central functions may be taken by the mammalian Ste-20-like protein kinase 1 (Mst1) (an evolutionarily conserved counterpart of yeast Ste-20 kinase [22]), p53 and Akt, also known as protein kinase B, the latter in parallel delaying further steps downstream on the pathway (figure 1, box). In this context, it was shown that hyperactivation of the Akt/PI3K/mTor signalling pathway by low serum stimulated nanotube formation in human malignant pleural mesothelioma [4] and this pathway inhibits Mst1 [22] and FOXO transcription factors [23]. p53 activation was found in astrocytes and neurons after TNT induction by H_2_O_2_ [24]. Downstream, further studies identified e.g. CDC42, the arp2/3 complex, myosin X, M-Sec, MHC class III protein Lst1, filamin, RalA-GTP, Ral binding protein 1 (RalBP1) and the exocyst complex as important regulators of TNT formation (for review, see [2]), proving the close interconnection with actin cytoskeleton-related processes.

The formed intercellular bridges, in the following specified as actin-based (AC)-TNTs, can finally be used to distribute various materials among cells of the connective, thereby counteracting the initial stress factors and opening discrete communication channels that are required for numerous body functions (for review, see [2]; figure 1*a*-3). This includes ‘passive’ diffusion of nutrients, smaller organelles, sterols, plasma membrane components, cytoplasmic and/or signalling molecules, proteins, RNAs, ions and so on, as well as the active, energy consuming, bidirectional transfer of organelles, protein complexes, etc., via myosin motors. Respective transfer and communication phenomena have already been described for a multitude of cargos and between a large number of cell types (for review, see [2]).

Nonetheless, diffusion as well as active transfer of molecules and organelles along AC-TNTs may be hampered by their actin backbone tightly enwrapped by the plasma membrane. Consistently, no mitochondria were observable inside AC-TNTs tensed between cultured PC12 cells [15], and also the diffusion of a dye with low molecular weight was found to be restricted [1]. However, such findings still have to be interpreted with caution, because *in vivo* the appearance and characteristics of TNTs might be significantly different. Also variances between cell types and developmental stages may exist, as well as mechanisms to match TNT permeability with current requirements. Such regulatory mechanisms are well known for plasmodesmata, where the size exclusion limit (SEL) can be varied, a mechanism that is discussed to be closely related to actin and microtubuli modifications [25] or callose homeostasis [26]. Likewise, alterations to the cytoskeleton and to the ECM would be efficient ways to influence TNT-based transfer processes between animal cells.

As, at least on the cell-culture level, AC-TNTs were frequently found to have limited lifetimes in the range of several minutes up to a few hours [2,15], the formed connective may rather reflect a temporary measure that does not permanently endanger cellular identity.

### 2.2. Stage 2: redox/metabolic rescue of cells under elevated reactive oxygen species levels

Within a tolerance range, based on the compensatory possibilities of AC-TNT-mediated cell connectivity, rising stress/ROS levels can be compensated for by increased nanotube formation (figure 2*a*-1). If, however, these levels further increase, endangering e.g. proper mitochondrial function, another TNT type will come into play (figure 1*b*-1). This type resembles so-called microtubule containing (MT)-TNTs [15], which differ from AC-TNTs by (i) an additional detyrosinated microtubule core, (ii) an increased diameter, (iii) a prolonged lifetime, and (iv) decreased membrane fluidity, suggested to be caused by the oxidation of unsaturated phospholipids [15]. Such ‘stabilized’ TNTs were first described in the context of an efficient rescue of apoptotic pheochromocytoma (PC12) cells, stressed by UV radiation, via nanotube-mediated mitochondria donation from unstressed control cells [15]. So far, it is unclear whether the increased lifetime is based on their detyrosinated microtubule backbone, on altered membrane fluidity, on ECM alterations or a combination thereof.

The mechanism involved in MT-TNT formation and their connection to AC-TNTs are still speculative. It was shown that microtubules polymerize from stressed cells towards the distal end of the nanotubes and microtubule depolymerization by nocodazol did not prevent their formation [15]. It is thus conceivable that pre-formed AC-TNTs are modified in response to exaggerated ROS levels. This transition is probably controlled by a pathway that connects increased stress/ROS levels and mitochondrial dysfunction with modifications of the microtubule cytoskeleton (figure 1, box). On the molecular level, the cascade may involve Mst1, the forkhead box O3 (FOXO3) transcription factor and the Bcl-2-like protein 11 (Bim), leading to mitochondrial depolarization by transitory mitochondrial outer membrane permeabilization [28], followed by the production of mitochondrial (m)ROS, cytochrome *c* release and caspase-3 activation [28], the latter providing negative feedback on Bim [29], temporarily preventing downstream apoptosis initiation (figure 1, box). In this context, it was shown that oxidative stress induces the activation of Mst1 and FOXO [30], e.g. during diabetes-induced hyperglycaemia [31]. AGE–RAGE interaction—at this stage—does not seem to play an essential role, because cells of RAGE(–/–) knockout mice as well as CHO cells are able to form TNTs under oxidative stress conditions [11,16], probably because ROS levels already exceed the necessary concentration. Consistently, in CHO cells the outgrowth of TNTs is undirected and A100A4 independent [16], implying that under these conditions TNTs can be formed also between pathological cells. It is important to note that moderate ROS production from plant mitochondria in response to oxidative stress increases the SEL of plasmodesmata and enhances intercellular trafficking [12], highlighting again the conceptual similarities across species.

The existence of a microtubule backbone inside MT-TNTs, along with their increased lifetime and diameter, represents an intercellular transit route allowing for efficient diffusion and active transfer within the collective. Now, also, larger organelles such as mitochondria can be transferred effectively via microtubule-specific motor proteins or intrinsic motor activities (figure 1*b*-2). Mitochondria inside TNTs have been observed for various cell types, e.g. between human endothelial progenitor cells and rat cardiomyoctes [32], human mesenchymal stem cells and cardiomyocytes and endothelial cells and cancer cells (summarized in [15]). Furthermore, it was shown that the mitochondrial Rho-GTPase Miro1, attaching mitochondria to motor proteins for antero- and retrograde transport, regulates their shuttling from mesenchymal stem cells to epithelial cells and its overexpression leads to increased stem cell repair [33]. The direction of such transfer processes might be defined by intercellular gradients, as exemplarily shown for the intracellular movement of mitochondria in yeast cells, linked with ATP gradients [34].

### 2.3. Stage 3: isolation and removal of ‘degenerated’ cells from the collective

It is a matter of course that, if stress/ROS levels further increase, tantamount with a failure of AC- and MT-TNT-based rescue mechanisms, cells will initiate apoptosis by following well-known pathways, here via mitochondria, involving mROS, cytochrome *c* and caspase-3 (figure 1*c*-1). This irreversible switch necessitates the break-down of remaining TNT connections in order to isolate and dispose of ‘degenerated’ cells from the collective and to prevent e.g. apoptotic signals from being transferred to ‘healthy’ cells (figure 1*c*-2). In this context, it was shown that the death signal Fas ligand can be propagated via TNTs between T lymphocytes to induce cell death [35,36], probably through activation of the caspase cascade.

The mechanism underlying the uncoupling of TNT connections—that presumably defines their lifetime too—so far is speculative. It could involve ‘passive’ scenarios, e.g. membrane rupture as a consequence of membrane ruffling, the remodelling of cytoskeletal components or cellular movements. However, the importance of this process rather implies the involvement of a precisely regulatable mechanism. In this context, cholesterol plays the essential role of regulating the physical properties of the plasma membrane by controlling lipid organization and phase behaviour and, thus, managing membrane fluidity and its mechanical strength [37]. In this context, cholesterol-rich lipid rafts were found to be enriched in TNT-forming mesothelioma as well as non-malignant mesothelial cells [38]. Changes in membrane composition, in particular the differential occurrence of cholesterol and its oxidized derivatives, the so-called oxysterols, in response to oxidative stress may cause dramatic changes in membrane fluidity modulating mechanical strength as well as the activity of membrane proteins, including enzymes and receptors [39]. Accordingly, studies have shown that cholesterol depletion from patient-derived primary cells by methyl-β-cyclodextrin strongly influenced TNT numbers [40]. Furthermore, depletion of cholesterol from the plasma membrane was demonstrated to lead to Akt inactivation and apoptosis [41]. Of note, Akt inactivation by methyl-β-cyclodextrin also reduces hyaluronan synthesis [42], which could again point to a participation of the ECM, e.g. as a stabilizing matrix.

In summary, membrane composition, in particular lipid and sterol homeostasis, in correlation with oxidative stress could play an essential role in TNT lifetime control. It is well documented that altered cholesterol/oxysterol homeostasis plays a key role in e.g. neurodegenerative diseases [43] and diabetes [44]. This would create opportunities to modulate the intercellular connectivity of cells or tissues, e.g. via pharmacological interventions, such as statin treatment, or targeted adaption of nutrition.

## 3. Physiological and pathological implications

It is evident that the ability of cells to increase their connectivity and to ‘share’ a common pool of resources represents a double-edged sword and requires effective control mechanisms. On the one hand, TNT-based supercellularity is a potent communication and rescue mechanism that provides significant survival advantages by allowing ‘individual’ cells—as part of a collective—to exchange information and to maintain redox and metabolic homeostasis under fluctuating physiopathological and environmental conditions.

In particular, the intercellular transfer of mitochondria has multiple beneficial functions. First, cells can be rescued from metabolic failures or mitochondrial dysfunction, i.e. ‘dead’ or impaired mitochondria disposed of by autophagocytosis can be replaced. In this context, it was found that the active transfer of mitochondria from adult mammalian stem cells to somatic cells rescued aerobic respiration in cells with non-functional mitochondria [45]. Similar observations were made for bone marrow-derived stromal cells protecting alveolar epithelium cells against acute lung injury [46] and mesenchymal stem cells rescuing injured cardiomyoblasts or endothelial cells [47]. Furthermore, the exchange of mitochondria can induce cell fate changes of stem or progenitor cells, or—more general—cellular reprogramming. In this view, it was suggested that nanotube-mediated transfer of mitochondria between adult human endothelial progenitor cells and neonatal rat cardiomyocytes could have a reprogramming function [32]. Similarly, it was shown that human mesenchymal stem cells reprogrammed adult cardiomyocytes towards a progenitor-like state through nanotube-mediated mitochondria transfer [48]. Finally, TNT-based supercellularity will have significant impact on the level of mitochondrial heteroplasmy and counteracts the evolutionary/developmental accumulation of deficient mitochondrial DNA (mtDNA) in otherwise isolated cells that can lead to severe diseases, like maternally inherited diabetes and deafness (MIDD) or mitochondrial myopathies. Accordingly, it was found that mesenchymal stem cells can transfer mitochondria to mtDNA-deficient cells and restore their mitochondrial function [49]. It is intriguing to note that, in a similar way at least, plastids—potentially also mitochondria—can be shuttled via plasmodesmata between plant cells, and their redox state regulates symplastic permeability [50].

On the other hand, TNT-based supercellularity can cause significant adverse side effects. It is obvious that anomalies in TNT formation and their mechanical or temporal stability will result in severe pathological implications. Current discussions regard neurodegenerative diseases, such as Parkinson's, Alzheimer's or Huntington's diseases, as closely related with (i) impaired mitochondrial function [51], (ii) oxidative stress [52], (iii) pathological cell–cell-communication [53] or (iv) intercellular prion propagation [54]. As the existence of TNTs between brain cells has already been shown (for review see [2]), the presented model describes a simple and compelling mechanism that melts together all four aspects into one common scenario. Accordingly, the degeneration of neurons would be explainable by reduced temporal and/or mechanical TNT stability or diminished TNT formation leading to a standstill in intercellular exchange, synonymous with a persistent undersupply and loss of networking capabilities, allowing the compensation of critical stress conditions. Alternatively, but not mutually exclusively, chronically elevated ROS levels could lead to a shift from AC- towards MT-TNT formation and apoptosis (figure 2*a*-2), accompanied by reduced stress tolerance, enhanced tissue damage or the risk of an uncontrolled intercellular spread of autoantigens and pathogens. Correspondingly, monomers and protofibrils of Aβ were detected in TNTs after extracellular uptake, and Aβ can be transferred via TNTs to induce cytotoxicity between e.g. astrocytes [24]. Further studies have shown that TNTs are able to transmit infectious forms of prion PRP^Sc^ between dendritic cells and primary neurons [55], and it is speculated that this transfer can occur via F-actin-dependent transport directed by myosin Va or lateral diffusion [56].

In another context, it was demonstrated that TNTs connect HIV-1-infected human T cells and present a novel route for virus transmission [57]. HIV-1 also induces TNT formation in human macrophages to promote virus propagation between cells [58,59]. Likewise, influenza A virus was shown to use actin-rich TNT structures to spread to neighbouring cells [60]. It is worth mentioning that in a similar way plasmodesmata are either ‘hijacked’ or manipulated by plant viruses for their intercellular spread, e.g. by increasing the SEL via actin cytoskeleton modifications [61]. It is interesting to add that for filopodial protrusions and TNTs, ‘surfing’ of pathogens such as viruses and bacteria along the outer membrane surface was also demonstrated (for reviews see [2,62]).

It should be borne in mind that the described scenarios not only apply to neurodegenerative diseases. Continued oxidative stress accompanied by chronic inflammation is one of the major characteristics of many chronic diseases, such as diabetes. It is known that AGE–RAGE interaction induces cROS that promotes mitochondrial superoxide generation specifically in hyperglycaemic environments [63], again indicative of a shift towards MT-TNT formation and apoptosis. Consistently, diabetic RAGE(–/–) knockout mice are protected from multiple diabetes-induced pathologies [64] and show reduced increases in mitochondrial superoxide e.g. in the renal cortex [63]. Also, metformin, a popular antidiabetic medication, inhibits AGE-induced renal tubular cell injury by suppressing ROS generation due to reduced RAGE expression [65]. Similarly, Mst1 was identified as a critical regulator of apoptotic β-cell death and function that under diabetogenic conditions is strongly activated and specifically induces the mitochondrial-dependent pathway of apoptosis through upregulation of Bim [66]. Consequently, knocking out Mst1 had a protective effect, characterized by abrogated caspase-3 cleavage, reduced cytochrome *c* release and lowered rate of apoptosis [66].

Also with regard to of cancer, the entanglement between oxidative stress and TNTs might play a pivotal role. Initiation and progression of a large number of cancers, such as breast or prostate cancer, have been linked to ROS [13]. At the same time, TNT formation between cancer cell lines and primary cancer cells derived from ovarian, breast, pancreatic, prostate and colon cancer was demonstrated and proposed to play an important role in cancer cell pathogenesis and invasion [4], achieved e.g. by an intercellular transfer of functional P-glycoprotein, a drug efflux pump mediating multi-drug resistance [67,68], or oncogenic microRNAs [69]. Furthermore, it has been shown that tumour cells can acquire mtDNA [70] or mitochondria displayed chemoresistance from the ‘host’ cells, as demonstrated for the TNT-dependent exchange of mitochondria between endothelial and cancer cells [71], implying that TNT-dependent genetic exchange can also lead to tumour heterogeneity [4]. Similarly, it was shown that tumour cells devoid of mtDNA can acquire mtDNA of host origin, resulting in stepwise recovery of respiration from primary to metastatic tumour cells [72]. This suggests that cancer cells, by forming TNT connections to preferentially—but not exclusively—senescent cells, will significantly increase their connectivity and chance of survival, whereas the prognosis for the patient deteriorates.

In consistency with the presented model, *in vivo* overexpression of S100A4 led to a significant increase in tumour growth and vascularization in a human melanoma xenograft M21 model [73]. Conversely, when silencing S100A4 by shRNA technology, a dramatic decrease in tumour development of the pancreatic MiaPACA-2 cell line was observed [73]. Furthermore, high levels of detyrosinated tubulin post-translational modifications were found to enhance disease aggressiveness of breast and prostate cancer [74], pointing to a participation of ‘stabilized’ MT-TNTs. This connection would provide an additional explanation for the mode of action of pharmacological compounds used for cancer treatment, such as microtubule-targeting agents (MTAs). These are known to suppress microtubule dynamics and to induce apoptosis by the mitochondrial-dependent pathway, via mROS, Bim and cytochrome *c* release [75]. Hence, they should significantly interfere with MT-TNT formation, depriving cancer cells of their survival strategy. Consistently, recent studies have shown that metformin can also suppress TNT formation between cancer cells [5]. A negative effect of oxysterols on TNT stability would be supported by the observation that they interfere with proliferation and cause the death of many cancer cells, such as glioblastoma, breast and prostate cancer, while they have little or no effect on senescent cells [76].

Given the above, the direct connection between ROS, TNT-based supercellularity and intercellular material exchange indeed represents a universal framework of numerous chronic and lifestyle diseases, and would mark these structures as highly promising targets for novel therapeutical and clinical approaches. Keeping in mind the suggested ubiquitous appearance, the structural and functional differences between AC- and MT-TNTs might in future enable precise control and manipulation of specific cell/tissue functions in order to balance pathological alterations.

## 4. Concluding remarks

The principal question of to what extent animal tissues represent continuous supercellular assemblies is anything but new. Already the late nineteenth century Camillo Golgi and Santiago Ramón y Cajal in disputed whether nerve cells in the brain represent a continuous reticular network or an assembly of ‘self-contained’ individuals. This dispute, against the background of TNTs observed between respective cells, might again become a topical issue.

Considering the striking structural and functional similarities among animal TNTs, plant plasmodesmata, fungal septal pores, bacterial nanotubes, etc. (figure 2*b*), the stipulating question arises as to whether these phenomena are expression of a conserved mechanism of cell collectives to maintain redox, metabolic and information homeostasis up to the organism level. It might be assumed that such a mechanism evolved as a necessary adaption of cellular life-forms to increasing O_2_ levels in the Earth's atmosphere approximately 2.5 billion years ago, requiring efficient strategies to compensate for oxidative stress or varying O_2_ and nutrient availability, in particular when considering the evolution of larger multicellular assemblies. In accordance with this view, it is speculated that e.g. plant cells interpret low concentrations of ROS as a stress that might be compensated for by increased cellular connectivity, while higher concentrations signal a hazardous state, where cellular isolation becomes beneficial [77]. An explanation for the relationship between mitochondria and increased symplastic transfer via plasmodesmata is that during anoxia enhanced intercellular connectivity would allow end products of the carbohydrate catabolism to flow from anoxic zones to regions capable of supporting oxidative phosphorylation, thus promoting efficient use of metabolic resources [78]. In this context, it is intriguing to note that membrane-derived nanotubes where also found between bacteria and being used for metabolic cross-feeding [79]. If, as suggested here, TNTs and other nanotube-based phenomena observed from plants down to bacteria are indeed homologous developments, the unbiased combination of current knowledge and the search for conserved or homologous functions, molecules and signalling pathways might help to boost our understanding of these poorly understood structures.

However, as general and appealingly simple the presented model may appear, its verification in the animal system puts us to the limits of detection methods and leads to a couple of elementary questions: how to prove functional TNTs in contiguous tissue or inside the body; and how to distinguish them from, *per definitionem,* close-ended filopodia, retraction fibres, tumour microtubes, streamers, cytonemes, etc., displaying a similar appearance (a question neglected by numerous ‘nano-/microtube’-related publications). How do we differentiate experimentally between intercellular transfer and communication phenomena based on TNTs and those mediated by e.g. gap junctions or extracellular shuttles, such as exosomes and argosomes, distributed by exo- and endocytosis? It is becoming increasingly obvious that corporate endeavours from different scientific fields, the development of novel tools and a unified terminology are desperately needed to prove or disprove the relevance of these peculiar structures in the *in vivo* situation.
